# Angiogenic Properties of Human Dental Pulp Stem Cells

**DOI:** 10.1371/journal.pone.0071104

**Published:** 2013-08-07

**Authors:** Annelies Bronckaers, Petra Hilkens, Yanick Fanton, Tom Struys, Pascal Gervois, Constantinus Politis, Wendy Martens, Ivo Lambrichts

**Affiliations:** Biomedical Research Institute, Hasselt University, Diepenbeek, Belgium; University of Southern California, United States of America

## Abstract

Angiogenesis, the formation of capillaries from pre-existing blood vessels, is a key process in tissue engineering. If blood supply cannot be established rapidly, there is insufficient oxygen and nutrient transport and necrosis of the implanted tissue will occur. Recent studies indicate that the human dental pulp contains precursor cells, named dental pulp stem cells (hDPSC) that show self-renewal and multilineage differentiation capacity. Since these cells can be easily isolated, cultured and cryopreserved, they represent an attractive stem cell source for tissue engineering. Until now, only little is known about the angiogenic abilities and mechanisms of the hDPSC. In this study, the angiogenic profile of both cell lysates and conditioned medium of hDPSC was determined by means of an antibody array. Numerous pro-and anti-angiogenic factors such as vascular endothelial growth factor (VEGF), monocyte chemotactic protein-1 (MCP-1), plasminogen activator inhibitor-1 (PAI-1) and endostatin were found both at the mRNA and protein level. hDPSC had no influence on the proliferation of the human microvascular endothelial cells (HMEC-1), but were able to significantly induce HMEC-1 migration *in vitro*. Addition of the PI3K-inhibitor LY294002 and the MEK-inhibitor U0126 to the HMEC-1 inhibited this effect, suggesting that both Akt and ERK pathways are involved in hDPSC-mediated HMEC-1 migration. Antibodies against VEGF also abolished the chemotactic actions of hDPSC. Furthermore, in the chicken chorioallantoic membrane (CAM) assay, hDPSC were able to significantly induce blood vessel formation. In conclusion, hDPSC have the ability to induce angiogenesis, meaning that this stem cell population has a great clinical potential, not only for tissue engineering but also for the treatment of chronic wounds, stroke and myocardial infarctions.

## Introduction

Stem cell biology has become an important field for the understanding of tissue regeneration and provides a very powerful tool in the treatment of traumatic injuries and various life-threatening pathological conditions, such as heart ischemia, spinal cord injury and Parkinson’s disease. Mesenchymal stem cells isolated from the bone marrow (BM-MSC) are the most intensively studied adult stem cells. They have been found to have clinical potential for gene therapy and for autologous repair of skeletal tissues, spinal cord injury and myocardial damages [1]–[3]. Since the procedure for harvesting BM-MSC is painful, invasive and causes complications in up to 30% of the cases, the search for alternative stem cell sources has been initiated [4]. More than a decade ago, Gronthos *et al.* demonstrated the existence of a MSC-like stem cell population within the dental pulp tissue [5]. These progenitor cells, designated human dental pulp stem cells (hDPSC), possess the capacity to differentiate into numerous cell types *in vitro* including odontoblasts [6], osteoblasts [5], [7], [8], chondroblasts [5], [7], [8], adipocytes [8], neuronal cells [9], [10] and even hepatocyte-like cells [11]. hDPSC have several advantages for clinical applications compared to other adult mesenchymal stem cells as they can be easily isolated from extracted adult teeth. Furthermore, these stem cells retain their multilineage differentiation capacity after cryopreservation, leading to the possibility to establish a stem cell bank [12]. In addition, recent studies indicate that hDPSC have a higher population doubling time, neural and epithelial stem cell properties than BM-MSC [13], [14].

The success of tissue engineering depends on oxygen and nutrient transport to the implanted cells. If blood vessel formation towards the transplanted tissue cannot be established rapidly, necrosis of the transplant will occur [15]. Therefore, in order to evaluate whether hDPSC are good candidates for tissue engineering, it is of utmost importance to investigate whether these stem cells possess the ability to induce angiogenesis. This knowledge is also required to gain more insight into the role of hDPSC in tooth formation and restoration. Angiogenesis is defined as the formation of new blood capillaries out of pre-existing blood vessels [16]. This type of blood vessel development is indispensable for physiological processes such as wound healing and reproduction. It is a complex, dynamic process involving following steps: degradation of the basement membrane and the extracellular matrix (ECM) surrounding the endothelial cells, endothelial cell proliferation and migration, tube formation, and maturation into functional blood vessels (e.g. recruiting mural cells). The molecular and cellular events during angiogenesis are tightly orchestrated by a delicate balance of numerous stimulatory and inhibitory signals including growth factors and their receptors, enzymes, matrix metalloproteinases (MMPs), cytokines, endogenous angiogenesis inhibitors, transcription factors, adhesion molecules and components of the ECM [16]–[19].

In cellular transplants, stem cells such as BM-MSC have been shown to promote angiogenesis in two distinct fashions: (1) the so-called paracrine effect by stimulating the formation of blood vessels from the host tissue through secretion of angiogenic factors or (2) by differentiating themselves into endothelial cells and thereby actively participating in the newly formed vascular structures [20]. Concerning paracrine induction of angiogenesis, hDPSC were previously shown to express several angiogenic factors such as vascular endothelial growth factor (VEGF), platelet-derived growth factor (PDGF) and fibroblast growth factor (FGF-2). Expression of these proteins was increased after injury [14], [21], [22]. Furthermore, hDPSC were able to induce the tube formation of human umbilical vein endothelial cells *in vitro* [22]. Several studies indicate that hDPSC also have angiogenic potential *in vivo*. Intramyocardial injection of GFP-transduced hDPSC in a rat model of myocardial infarction, resulted in an improvement in cardiac function, reduction of the infarct size and a higher neovascularization in cell-treated animals compared to controls [23]. Transplantation of a CD31-negative side population of porcine DPSC in a mouse hindlimb ischemia model produced a higher blood flow and capillary density compared to animals treated with bone marrow and adipose CD31- MSC [24]. Nevertheless, the precise cellular and molecular mechanisms by which hDPSC contribute to blood vessel formation, remain to be illuminated. In the present study, we further elucidated the paracrine effects of hDPSC on angiogenesis. Firstly, the angiogenic factors secreted by hDPSC were identified by means of a protein array that allows the simultaneous detection of 55 different angiogenesis-related proteins. These results were validated by RT-PCR, ELISA and *in situ* immunohistochemistry. Secondly, we studied the *in vitro* effect of hDPSC on endothelial proliferation and migration, two essential steps of angiogenesis. Finally, we assessed the ability of hDPSC to induce the formation of fully functional blood vessels in the chicken chorioallantoic membrane (CAM) assay.

## Materials and Methods

### Cell Isolation and Culture

Human third molars were collected with written informed consent from patients (15–20 years of age) undergoing extraction for orthodontic or therapeutic reasons at Ziekenhuis Maas en Kempen, Bree, Belgium. Written informed consent of underaged patients was obtained via their guardians. This study was approved by the medical ethical committee of Hasselt University. Dental pulp tissue was obtained with forceps after mechanically fracturing the teeth with surgical chisels. Human dental pulp stem cells (hDPSC) were isolated from the pulp tissue by means of the outgrowth method as previously described [8], [25], [26]. The cells were maintained in Minimal essential medium, alpha modfication (αMEM, Sigma, St-Louis, MO) supplemented with 10% fetal bovine serum (FBS, Biochrom AG, Berlin, Germany), 2 mM L-glutamine, 100 U/ml penicillin and 100 µg/ml streptomycin (Sigma). All hDPSC were routinely screened for mesenchymal stem cell markers CD29, CD44, CD90, CD105 and CD146. hDPSC were not pooled, but in each experiment hDPSC of at least 3 different donors from passage 2–5 were used.

The human microvascular endothelial cell line 1 (HMEC-1) was purchased from the Centre for Disease Control and Prevention (Atlanta, GA). HMEC-1 were grown in MCDB 131 medium (Invitrogen, Carlsbad, CA) containing 10% FBS, 10 ng/ml human epidermal growth factor (hEGF, Gibco, Paisley, UK), 1 µg/ml hydrocortisone (Sigma), 10 mM L-glutamine (Sigma), 100 U/ml penicillin and 100 µg/ml streptomycin. Mouse Brain endothelial cells (MBEC) were kindly provided by Prof. Dr. S. Liekens (Leuven, Belgium) and maintained in DMEM Glutamax™ I (Invitrogen) supplemented with 10% FBS, 100 U/ml penicillin and 100 µg/ml streptomycin, as described earlier [27], [28].

HMEC-1, MBEC and hDPSC were grown at 37°C in a humidified atmosphere with 5% CO_2_ and the medium was changed every 2–3 days.

### Collection of Conditioned Medium (CM) and Cell Extracts

Conditioned medium (CM) was made by seeding hDPSC at a density of 20,000 cells/cm^2^ in 25 cm^2^ cell culture flasks. Cells were allowed to adhere overnight, washed twice with PBS and incubated with 5 ml of αMEM containing 0.1% FBS. 48 hours later, the medium was collected and stored at −80°C. Next, cells were washed with PBS and lysed using cell lysis buffer (RIPA buffer containing ‘mini protease inhibitors’ (Roche, Basel, Switzerland)). After an incubation on ice for 10 minutes, the cell mixture was centrifuged at 9000 g at 4°C. Supernatant (containing the proteins) was aliquoted and stored at −80°C. Protein content was determined with a BCA assay (Thermo Fischer Scientific, Rockford, IL).

### Antibody Array

In order to identify angiogenic factors in the cell extracts and conditioned medium (CM) of hDPSC, an antibody array (Proteome Profiler™ Human Angiogenesis Antibody Array, R&D systems, Minneapolis, MN) was performed according to the manufacturer’s instructions. Briefly, the array membranes were incubated with a blocking buffer for 1 h. While the membranes were blocking, CM or cell extracts (with a protein content of 1 mg/ml) were incubated with detection antibodies for 1 h at room temperature. Next, sample/antibody mixtures were added to the membranes and incubated overnight at 4°C on a rocking platform. Following several wash steps, membranes were incubated with HRP-conjugated streptavidin for 30 min. Next, the membranes were washed again and chemiluminescent detection reagents (ECL plus, GE Healthcare, Little Chalfont, UK) were added sequentially. Light signals were detected by exposing the membranes to an X-ray film for 2 minutes. Quantification of the dots was performed with Image J dot blot analyzer software.

### Enzyme-linked Immunosorbent Assay (ELISA)

ELISAs were performed on hDPSC CM or cell extracts (10 µg total protein/ml) to determine the concentration of certain angiogenic factors and to confirm the semi-quantitative results of the antibody array. Following ELISA kits were used according to the manufacturers’ guidelines: FGF-2 (Biolegend, San Diego, CA), Endostatin (Raybiotech, Norcross, GA), IGFBP-3 (Raybiotech), IL-8 (Biolegend), MCP-1 (Peprotech, Rocky Hill, NJ), PAI-1 (Invitrogen), TIMP-1 (Peprotech), uPA (Boster Biological Technology, Encyclopedia Circle Fremont, CA ), VEGF (Raybiotech). Unless stated otherwise, ELISAs were performed on conditioned medium or cell extracts of at least 6 different donors.

### Reverse transcriptase Polymerase Chain Reaction (RT-PCR)

Total RNA was extracted from hDPSC using the RNeasy Plus Mini kit (Qiagen, Venlo, The Netherlands) which includes an additional step to remove genomic DNA. Concentration and purity of the extracted RNA was determined by measuring the optical density at 260 nm and the 260/280 nm ratio. 700 ng of total RNA was reverse-transcribed to cDNA in a total reaction mixture of 20 µl with the Reverse Transcription System (Promega, Leiden, The Netherlands). Next, polymerase chain reaction was performed using the following reaction mixture: 1 µM of forward/reverse primer (Eurogentec, Seraing, Belgium), 200 µM of dNTPs and 0.75 U of Taq Polymerase (Roche). The thermal cycle parameters were: denaturation for 5 min at 94°C, followed by 35 cycles of denaturation (60 s at 95°C), annealing (60 s at a primer-specific temperature) and an elongation step (45 s at 72°C), and finally an additional elongation step of 10 min at 72°C. Housekeeping genes β-actin, GusB and β2-microglobulin were used as a control for the PCR reaction. All forward and reverse primer sequences, their product size and melting temperature (T_m_) are presented in Table 1.

**Table 1:** RT-PCR primers.

Gene	Primer	Sequence	Product size (bp)	Tm (°C)
**Angiogenic factors**				
**Endostatin**	Forward	ATG-CTG-ACA-TTC-ACC-TGC-C	174	58
	Reverse	ATG-AAG-TCA-GCA-CCT-GCT-GG		62
**HGF**	Forward	TTC-ATG-ATG-TCC-ACG-GAA-CA	575	58
	Reverse	TTG-TAT-TGG-TGG-GTG-CTT-CA		58
**IGFBP-3**	Forward	TTG-CAC-AAA-AGA-CTG-CCA-AG	275	58
	Reverse	CAA-CAT-GTG-GTG-AGC-ATT-CC		60
**IL-8**	Forward	AGG-GTT-GCC-AGA-TGC-AAT-AC	420	60
	Reverse	AAA-CCA-AGG-CAC-AGT-GGA-AC		60
**MCP-1**	Forward	AAG-CAG-AAG-TGG-GTT-CAG-GA	300	60
	Reverse	GCA-ATT-TCC-CCA-AGT-CTC-TG		60
**PAI-1**	Forward	ATA-CTG-AGT-TCA-CCA-CGC-CC	320	62
	Reverse	GTG-GAG-AGG-CTC-TTG-GTC-TG		64
**TIMP-1**	Forward	GCT-TCT-GGC-ATC-CTG-TTG-TT	462	60
	Reverse	TTT-GCA-GGG-GAT-GGA-TAA-AC		58
**uPA**	Forward	GCC-ATC-CCG-GAC-TAT-ACA-GA	417	62
	Reverse	AGG-CCA-TTC-TCT-TCC-TGG-GT		60
**VEGF**	Forward	CCT-TGC-TGC-TCT-ACC-TCC-AC	280	64
	Reverse	ATC-TGC-ATG-GTG-ATG-TTG-GA		58
**Housekeeping genes**				
**β-actin**	Forward	AAA-TCT-GGC-ACC-ACA-CCT-TC	185	56
	Reverse	AGA-GGC-GTA-CAG-GGA-TAG-CA		56
**β2-Microglobulin**	Forward	CTC-ACG-TCA-TCC-AGC-AGA-GA	213	56
	Reverse	CGG-CAG-GCA-TAC-TCA-TCT-TT		56
**Gus B**	Forward	AGC-CAG-TTC-CTC-ATC-AAT-GG	160	56
	Reverse	GGT-AGT-GGC-TGG-TAC-GGA-AA		56

### Immunohisto- and Immunocytochemical Staining

For immunohistochemistry, human dental pulp tissues were fixed in 4% paraformaldehyde (PFA). Next, tissues were dehydrated in graded ethanol, embedded in paraffin and serially sectioned at 7 µm. After rehydration, sections were incubated in 10% antigen retrieval solution (Dako, Glostrup, Denmark) and heated 3 times with a microwave at 480 W for 5 minutes with pauses of 2 minutes. Next, tissue sections were allowed to cool down for 30 minutes and were incubated with 10% normal donkey serum to prevent non-specific binding of the antibodies. After a wash with PBS, sections were incubated overnight on 4°C with primary antibodies (see Table 2). After 3 washes of 5 minutes in PBS, an Alexa fluor® 555 (red)-anti-mouse or an Alexa fluor® 488 (green)-labeled anti-rabbit secondary antibody (Invitrogen) were added for 30 minutes. Cell nuclei were counterstained with DAPI and then slides were mounted with anti-fade mounting medium (Dako). Fluorescence was visualized with a Nikon Eclipse 80 i Fluorescence microscope equipped with a Nikon DS-2 MB Wc digital camera (Nikon Tokyo, Japan). Stainings in which primary antibodies were omitted, were used as a control.

**Table 2:** Primary antibodies for immunohistochemistry.

Antigen	Type	Company	Dilution
**CD146**	Rabbit monoclonal	Abcam	1/100
**FGF-2**	Mouse monoclonal	Sigma	1/250
**VEGF**	Mouse monoclonal	R&D systems	1/50
**MCP-1**	Mouse monoclonal	R&D systems	1/100

### Cell Proliferation Assay

The effect of hDPSC CM on endothelial cell proliferation was assessed with a 3-(4, 5-dimethylthiazolyl-2)-2,5-diphenyltetrazolium bromide (MTT) assay. MBEC or HMEC-1 were seeded in a 96-well plate at a density of 15,625 cells/cm^2^. The next day, cells were washed twice with PBS and the medium was replaced with CM of hDPSC. αMEM with 10% FBS or 0.1% FBS was used as positive or negative control respectively. 72 h later, the media were removed and 500 µg/ml MTT (Sigma) was added to the wells. The MTT solution was removed after 4 h of incubation at 37°C. At each well, a mixture of 0.01 M glycine and DMSO (Sigma) was added. The absorbance was measured at a wavelength of 540 nm with a Benchmark microplate reader (Biorad Laboratories).

### Transwell Migration Assay

The ability of hDPSC to affect the migratory behavior of endothelial cells was assessed by means of a transwell migration assay. hDPSC were seeded at a density of 50,000 cells/cm^2^ in a 24-well plate in αMEM with 10% FBS (lower compartment). Cells were allowed to adhere for 24 hours after which they were washed and incubated with αMEM with 0.1% FBS for 24 hours to induce secretion of angiogenic factors. Next, HMEC-1 were seeded on Thincert™ tissue culture inserts (pore size 8 µm; Greiner Bio-One, Frickenhausen, Germany) in αMEM with 0.1% FBS at a density of 150,000 cells per cm^2^ (upper compartment). The inserts were placed in the wells with hDPSC, allowing diffusion of soluble factors which attract the HMEC-1 of the upper compartment towards the lower compartment. Lower wells filled with αMEM with 10% FBS or 0.1% FBS served as positive and negative controls respectively. In some conditions, antibodies against anti-VEGF (R&D, 500 ng/ml) and anti-MCP1 (R&D, 1 µg/ml) or a combination of both were also added to the hDPSC. In other experiments, the PI3K-inhibitor LY294002 (Sigma, 10 and 1 µM) and the MEK-inhibitor U0126 (Sigma, 10 and 1 µM) were added to the upper compartment containing the HMEC-1. 24 hours later, HMEC-1 transmigrated towards the lower surface of the filter were fixed with 4% PFA and stained with 0.1% crystal violet in 70% ethanol for 10 minutes and washed with PBS. Cells were photographed in 2 random microscopic fields per well using a Nikon eclipse TS100 inverted microscope with a Jenoptik ProgRes C3 camera (Jenoptik, Jena, Germany) at a total magnification of 100x. Quantification was performed by means of Carl Zeiss Axiovision 4.6 Software (Carl Zeiss Vision, Aalen, Germany) and values were expressed as mean area percentage.

### Chicken Chorioallantoic Membrane Assay

Fertilized chicken eggs (*gallus gallus*) were incubated for 3 days at 37°C at constant humidity. At day 3 of embryonic development, albumin (3–4 ml) was removed to detach the egg shell from the developing chorioallantoic membrane (CAM) and a 1 cm^2^ window was opened in the shell exposing the CAM. The window was covered with cellophane tape and the eggs were returned to the incubator. At E9, 50,000 hDPSC were resuspended in 30 µl growth factor reduced matrigel (BD, Franklin lakes, NJ) and allowed to solidify at 37°C for at least 2 hours. Pure matrigel droplets of 30 µl served as negative controls. The droplets were applied onto the CAM while placement onto big pre-existing blood vessels was avoided. Next, the windows were covered and the eggs were incubated for 3 more days. At E12, the CAM was removed and angiogenesis was assessed. Pictures of each CAM were taken under a stereomicroscope (Wild M3Z Stereomicroscope, HEERBRUGG, Heerbrugg, Switserland) using a digital camera (Nikon DN100 Digital Net Camera). All vessels intersecting two concentric circles (with a radius of 1.5 and 2 mm) digitally positioned around the matrigel droplets were counted double blind and independently by two investigators.

### Statistical Analysis

Quantitative results are presented as mean ± standard deviation (S.D), unless stated otherwise. Data were analyzed using Graphpad Prism 5 Software (Graphpad, San Diego, CA). First, data were analyzed for normality using D’Agostino & Pearson omnibus normality test. To compare 2 different experimental groups (e.g. CAM assay) following statistical tests were used: if data were Gaussian distributed, p-values were calculated using an unpaired student t-test. Otherwise, a non-parametric Mann-Whitney U test was employed. When multiple groups were compared a one-way ANOVA test with Bonferroni Multiple Comparison Post-hoc test was used for Gaussian distributed data. In case of non-parametric data, a Kruskal-Wallis test with Dunn’s Post-hoc test was performed. Any p-value <0.05 was considered to be statistically significant.

## Results

### Expression of various Angiogenesis-related Molecules

**Figure 1:**
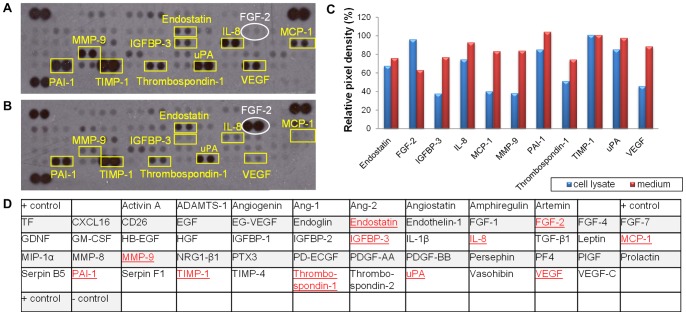
Antibody array on both conditioned medium (A) and cell lysates (B) of hDPSC. 48 hour conditioned medium and cell lysates were prepared from hDPSC. Angiogenesis-related proteins present were identified with an antibody array (A, conditioned medium; B, cell lysates). Graph C indicates the relative expression of each factor while table D shows the location of the antibodies spotted on the human angiogenesis antibody array. In red the proteins that were detected in the CM or in the cell lysates. Numerous pro-angiogenic factors such as VEGF and MCP-1 but also anti-angiogenic factors such as endostatin were found in both CM and cell lysates while FGF-2 was only present in the cell lysate. This assay was performed twice with CM and cell lysates of two different donors.

**Figure 2:**
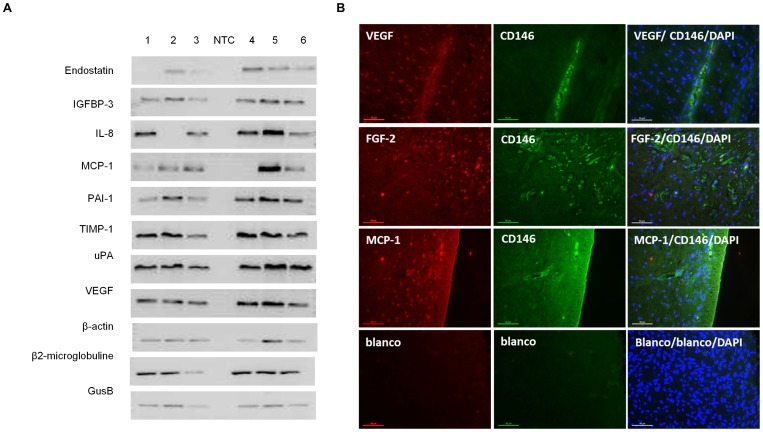
A) RT-PCR confirmation of the expression various angiogenesis related molecules. mRNA was isolated from hDPSC of 6 different donors and RT-PCR was performed. Housekeeping genes β-actin, GusB and β2-microglobulin were used as a control for the PCR reaction. All donors expressed VEGF, IGFBP-3, uPA, PAI-1 and TIMP-1, while endostatin, IL8 and MCP-1 where found in 5 of the 6 donors. B) Immunofluorescence (IF) on whole pulp tissue. Dental pulp tissue of 4 different donors were fixed in 4% PFA, embedded in paraffin and 7 µm sections were made. Subsequently, immunofluorescent stainings were performed with antibodies against the angiogenic factors (VEGF, MCP-1 and FGF-2, red) and against the endothelial cell and hDPSC marker CD146 (green). Nuclei were counterstained with DAPI (blue). Pictures show a representative staining of one donor. It is very clear that the dental pulp cells are slightly positive for CD146, while the endothelial cells that form the walls of the blood vessels express high amounts of CD146. Furthermore, almost all cells present within the human dental pulp tissue were found to express VEGF, MCP-1 and FGF-2 *in situ*. Scale bars = 50 µm.

A human angiogenesis array was performed to study the relative expression of 55 different angiogenesis-related proteins in both the cell lysates and the CM of hDPSC (Figure 1). A wide variety of pro-angiogenic factors such as vascular endothelial cell growth factor (VEGF), interleukin-8 (IL-8) and monocyte chemotactic protein-1 (MCP-1) were detected in both conditions. As shown by RT-PCR (Figure 2A), VEGF was found in all 6 donors, while MCP-1 and IL-8 were detected in only 5 out of 6 donors. ELISA on CM revealed that VEGF and MCP-1 were abundantly secreted by the hDPSC at a concentration of respectively 2403 and 233 pg/ml (Table 3). *In situ* immunohistochemistry of dental pulp tissue revealed the presence of VEGF and MCP-1 (Figure 2B), leading to the conclusion that this expression is not induced by culture conditions. The human angiogenesis array clearly showed that fibroblast-growth factor-2 (FGF-2) was only present in the cell lysate and not in the CM. This was validated by means of ELISA, which showed that FGF-2 was present in the cell lysates at a concentration of 613±114 pg/ml while the amount of FGF-2 secreted into the medium was below the detection range of the ELISA kit (Table 3). Immunofluorescence also showed FGF-2 expression in dental pulp tissue (Figure 2B).

**Table 3:** Concentration of various angiogenesis related factors in the conditioned medium of hDPSC (as determined by ELISA).

Pro-or anti-angiogenic factor			Concentration (pg/ml)
**Endostatin**		CM	271±42
**FGF-2**	**Fibroblast growth factor 2**	CM	Below detection limit
		Cell lysate	613±114 pg/ml
**IGFBP3**	**Insuline-like growth factor binding protein-3**	CM	656±97
**IL-8**	**Interleukin-8**	CM	25±9
**MCP-1**	**Monocyte chemotactic protein 1**	CM	233±49
**PAI-1**	**Plasminogen activator inhibitor-1**	CM	2408±536
**TIMP-1**	**Tissue inhibitors of metalloproteinase -1**	CM	7708±920
**uPA**	**Urokinase-type plasminogen activator**	CM	1368±649
**VEGF**	**Vascular endothelial cell growth factor**	CM	2403±282

Values are represented as mean ± S.E.M. (n≥3). CM = conditioned medium.

In addition, both the angiogenesis array and RT-PCR demonstrated the expression of numerous enzymes such as urokinase plasminogen activator (uPA), tissue inhibitor of metalloproteinase (TIMP-1), and plasminogen activator inhibitor-1 (PAI-1) (Figure 1 and 2). The concentration of PAI-1, TIMP-1 and uPA in the hDPSC CM was 2408±536, 7708±920 and 1368±649 pg/ml respectively (Table 3). Furthermore, the endogenous angiogenesis inhibitors endostatin and insulin-like growth factor-binding protein 3 (IGFBP-3) were detected in CM at a concentration of 271±42 and 656±97 pg/ml respectively.

### Effect of hDPSC on Endothelial Cell Proliferation

**Figure 3:**
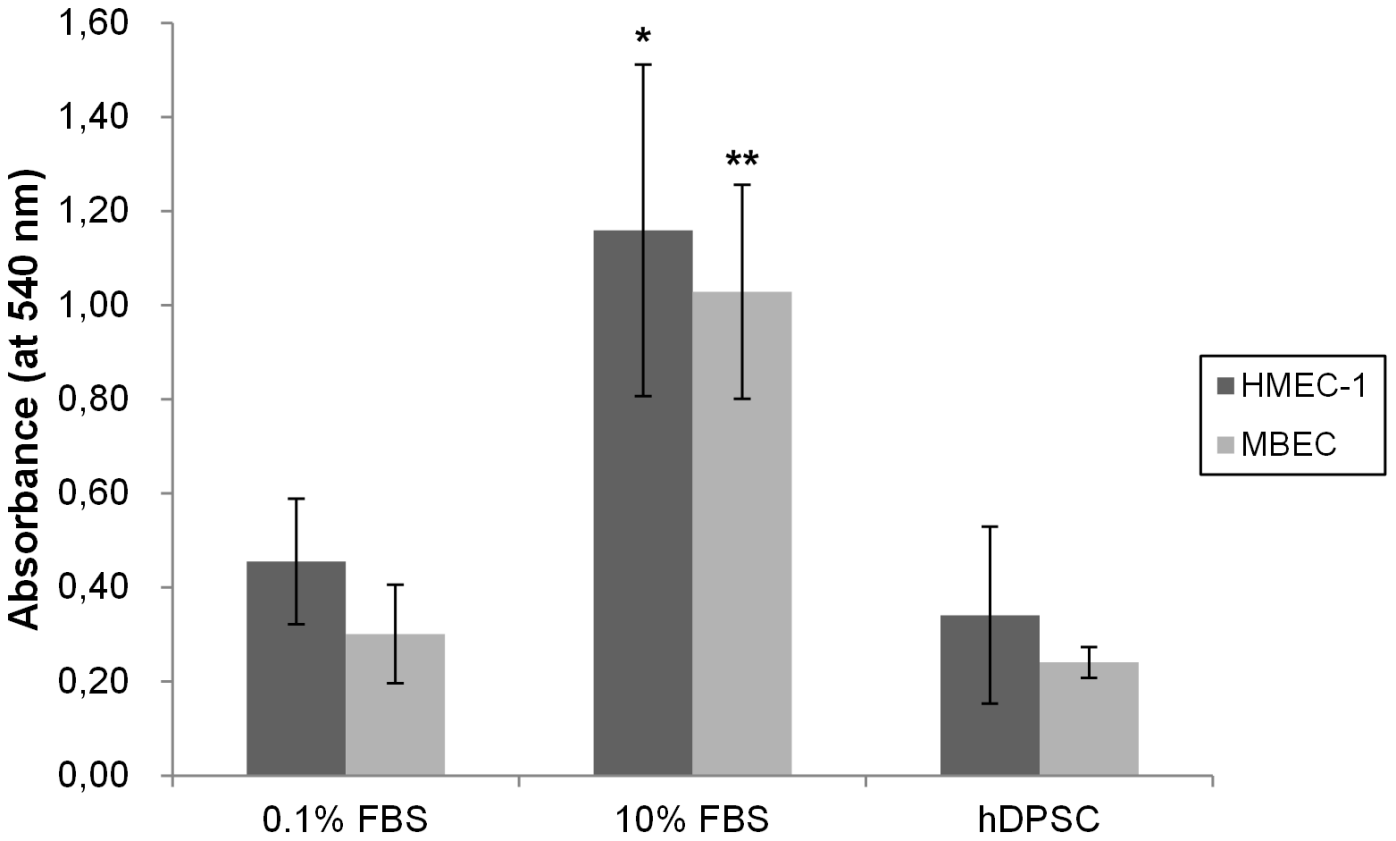
Cell proliferation (MTT) assay: MBEC or HMEC-1 endothelial cells were seeded in 96-well plates and 24 hours later the CM of hDPSC was added. αMEM containing 10% FBS was included in the study as a positive control. 72 h later, the media were removed and cell proliferation was assessed. Conditioned medium of hDPSC was not able to increase the cell growth of neither MBEC nor HMEC-1 compared to control medium (with 0.1% FBS). Incubation of αMEM with 10% FBS resulted in a 3-fold increase in cell growth in both MBEC and HMEC-1. This assay was repeated 3 times with a total number of at least six different donors; *p<0.05; **p<0.01.

In order to assess whether a particular (stem) cell population possesses angiogenic activity, it is not only necessary to examine the angiogenic proteins these cells produce, but also to explore the effect of these cells on the behavior of endothelial cells (EC). The effect of hDPSC on EC proliferation was tested by means of an MTT assay (Figure 3). hDPSC CM had no significant effect on the proliferation of MBEC and HMEC-1 compared to the negative control condition (αMEM with 0.1% FBS). Addition of αMEM with 10% FBS resulted in an 3-fold increase in absorbance and thus caused a significant cell growth of both cell types.

### Effect of hDPSC on EC Migration

**Figure 4:**
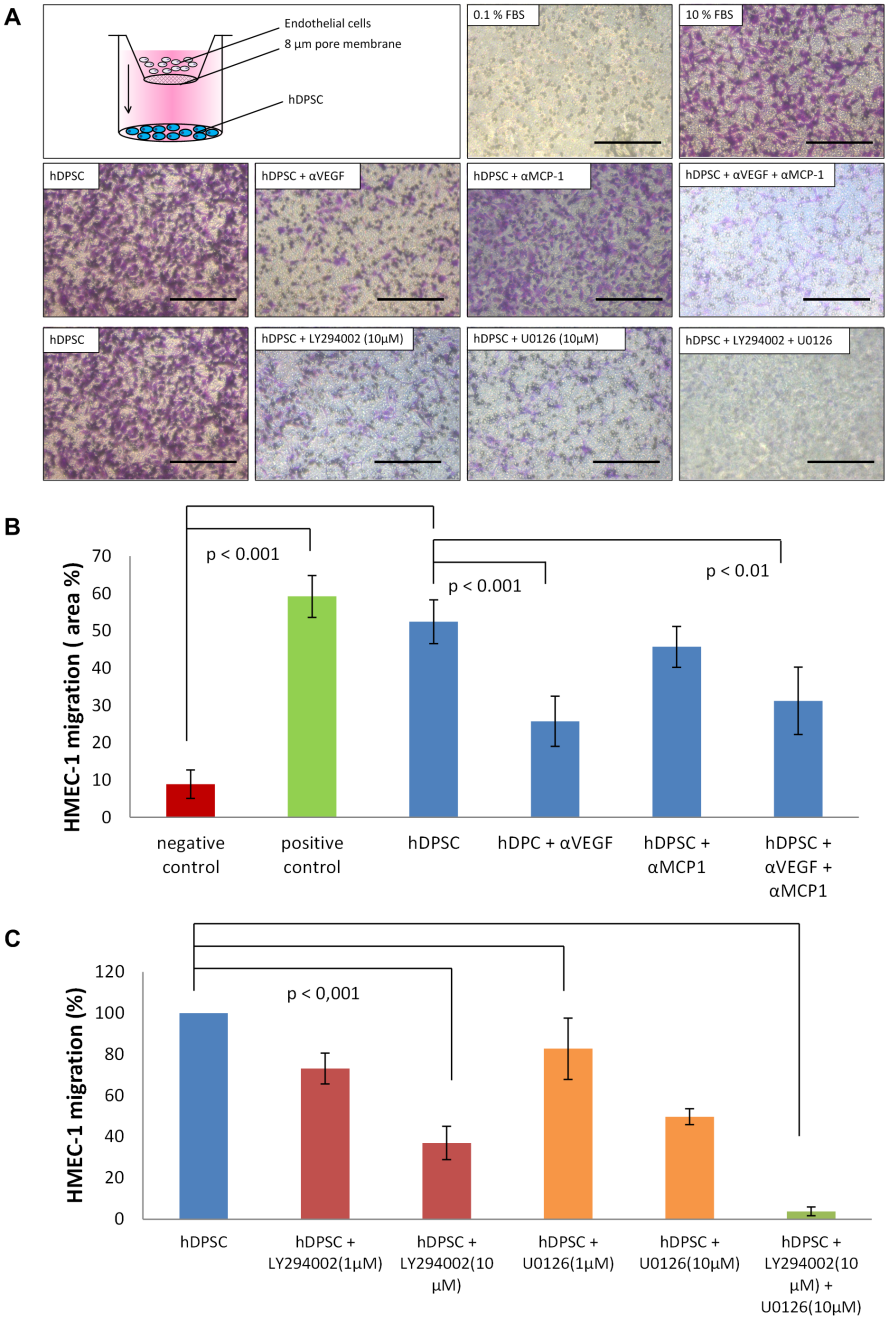
Transwell migration assay: hDPSC significantly induced the migration of the endothelial cells HMEC-1. A) Transwell system: hDPSC were seeded in the lower compartment. The next day, cells were put on α-MEM 0.1% FBS. 24 h later, the cell culture inserts with a filter (with a pore size of 8 µm) containing HMEC-1 were placed onto the wells with hDPSC. 24 h later, migration was assessed by fixing the cells in 4% PFA, staining with 0.1% crystal violet and pictures were taken. Scale bars = 200 µm. B) Graph showing the effect of different conditions on the HMEC-1 proliferation (Y-axis = the area occupied by violet stained HMEC-1, in %). hDPSC significantly (p<0.001) induced HMEC-1 migration; This migration could be partially inhibited by addition of anti-VEGF antibodies but not by addition of MCP-1 antibodies. C) Graph presenting the effect of PI3K-inhibitor LY294002 and the MEK-inhibitor U0126 (10 and 1 µM) on hDPSC-induced HMEC-1 migration. (HMEC-1 migration caused by hDPSC was set at 100%). Both LY294002 and U0126, were able to significantly decrease the hDPSC-induced HMEC-1 migration at a concentration of 10 µM. Addition of only 1 µM of these inhibitors, slightly reduced the migration, but this reduction was not statistically significant. When both LY294002 and U0126 at a concentration of 10 µM each were added, the transwell migration was completely inhibited. This experiment was performed 5 times with hDPSC of at least 5 different donors.

As EC migration is an essential step in angiogenesis, it was examined whether hDPSC are able to induce the migration of endothelial cells (HMEC-1) by means of a transwell migration assay (Figure 4). HMEC-1, seeded onto a 8µm insert, significantly migrated towards hDPSC, seeded in the well beneath, after an incubation period of 24 h (p-value <0.001). This effect could be inhibited for 50% by adding antibodies against VEGF. The addition of anti-MCP-1 antibodies had no influence on the hDPSC-induced migration. When both MCP-1 and VEGF-1 antibodies were added to the HMEC-1, this also lead to a decrease of 50%, which is probable due to the inhibitory of the anti-VEGF antibody alone. To elucidate the intracellular pathways within the HMEC-1 involved in the migration-inducing capacities of hDPSC, the PI3K-inhibitor LY294002 (10 and 1 µM) and the MEK-inhibitor U0126 (10 and 1 µM) were added to the upper compartment containing the HMEC-1. Both molecules could significantly reduce the hDPSC-induced HMEC-1 migration at a concentration of 10 µM. LY294002 decreased the migration for 60%, while U0126 prevented the migration for 50%. Addition of only 1 µM of these inhibitors, slightly reduced the migration, but this reduction was not statistically significant. When both LY294002 and U0126 (10 µM each) were added, HMEC-1 migration was completely stopped.

### Chicken Chorioallantoic Membrane Assay

**Figure 5:**
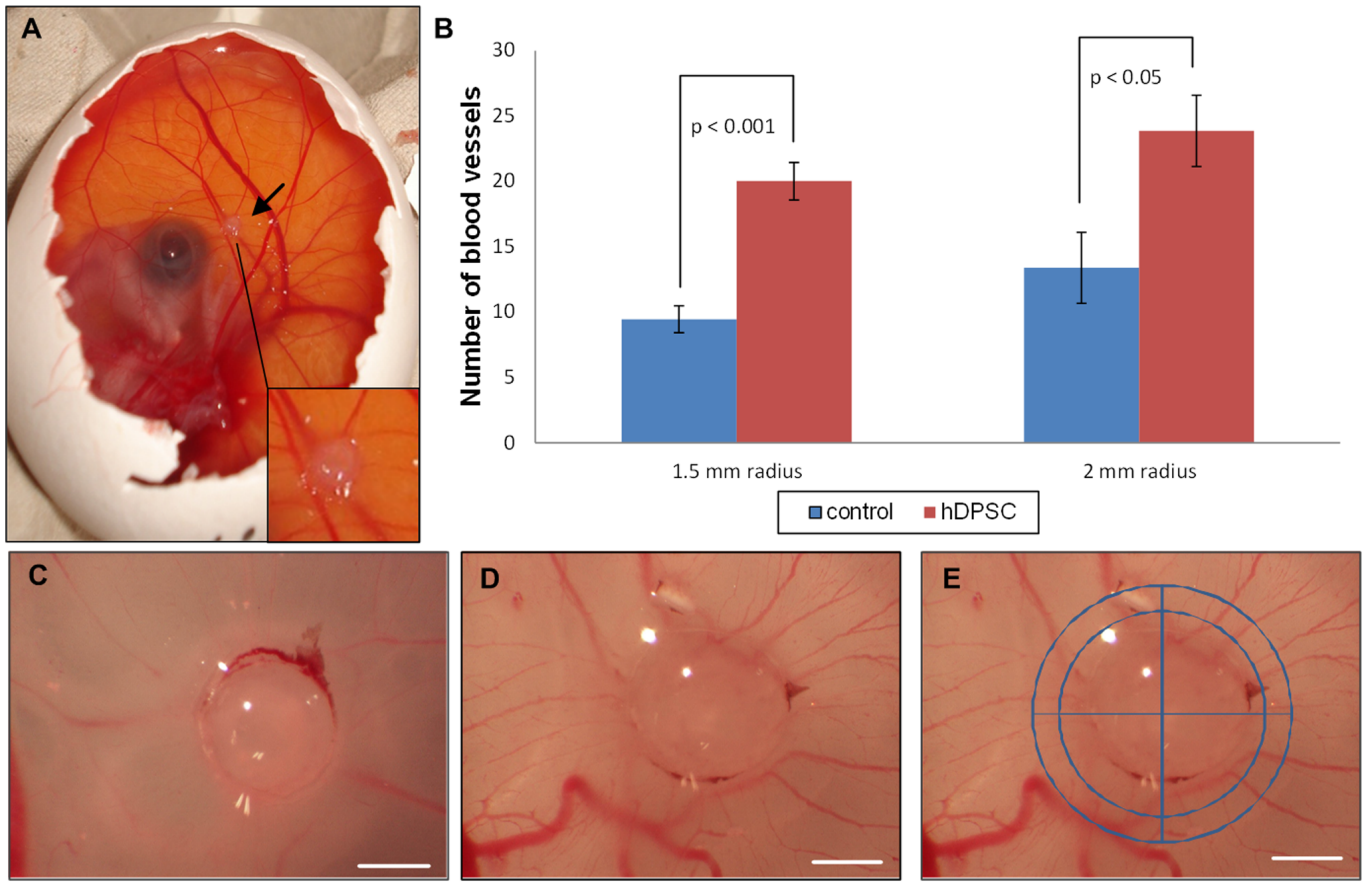
Chorioallantoic membrane (CAM) assay. At day 9 of embryonic development, matrigel droplets with or without 50,000 hDPSC were applied onto the CAM of a chicken embryo (black arrow, Figure 5a). 72 hours later, pictures were taken (figure 5c–e). Figure 5c is a control matrigel droplet, while Figure 5d is a droplet containing 50,000 hDPSC. White scale bars represent 1 mm. To assess angiogenesis, two circles (with a radius of 1.5 and 2 mm, see figure 5e), were digitally positioned around the matrigel droplets and the blood vessels intersecting these circles were counted double blind. Graph 5b: hDPSC were able to significantly increase the number of capillaries intersecting both circles. This assay was repeated 4 times with hDPSC of 4 different donors to gain a database of at least 27 individual eggs. Values are represented as means ± S.E.M.

A CAM assay was performed to provide more insights into the angiogenic properties of hDPSCs *in ovo* (Figure 5a). After incubating the eggs for 3 days with hDPSC-containing matrigel droplets, all vessels intersecting two concentric circles (radius 1.5 and 2 mm) digitally positioned around the droplets were counted in a double-blinded fashion (Figure 5e). The number of blood vessels intersecting both circles was significantly higher in hDPSC-containing matrigel droplets compared to control conditions, meaning that hDPSC are able to significantly induce angiogenesis in the CAM assay (Figure 5b–d). The allantoic vessels grew radially towards the hDPSC-containing matrigel droplets and thus the typical spoke wheel pattern was observed (Figure 5d). [29].

## Discussion

The delivery of autologous stem cells to increase angiogenesis is an emerging treatment option for pathologies associated with insufficient vascularization such as chronic wounds, stroke and myocardial infarctions [20]. With regard to tissue engineering, angiogenesis also plays an important role as the formation of new blood vessels towards transplanted cells is a rate-limiting process. This stresses an urgent need for an easily accessible stem cell source with a high proliferation rate, that can also provide sufficient cell numbers for transplantation. Within the human tooth, a mesenchymal-like stem cell population has been discovered, which can be easily isolated, cultured and differentiated into various cell types such as osteoblasts, odontoblasts, chondrocytes, neuron-like cells and even hepatocyte-like cells [5]–[11]. The objective of this study was to examine whether these stem cells, namely hDPSC, are able to induce angiogenesis and to elucidate the regulatory pathways involved in their potential pro-angiogenic actions.

Transplanted stem cells are considered to contribute to blood vessel formation by two distinct mechanisms: (1) the so-called paracrine effect: by secreting angiogenic factors that induce angiogenesis from host endothelial cells and (2) by differentiating themselves into endothelial cells and thus actively incorporating into the walls of blood vessels [20]. Concerning the endothelial differentiation potential of hDPSC, several studies report that incubation of hDPSC *in vitro* with specialized differentiation media resulted in an upregulation of EC markers like von Willebrand factor (vWF), CD31, CD34 and CD105 in a (sub)population of the cells [30]–[32]. In addition, stem cells of exfoliated teeth (SHED) were shown to differentiate into endothelial-like cells *in vivo* when seeded in biodegradable scaffolds and transplanted into immunodeficient mice [33]. Regarding the paracrine angiogenic effects of hDPSC, several studies have indicated the expression of certain angiogenic factors by these stem cells, such as VEGF, FGF-2, PDGF, MMP-9, IGF-1 and TGF-β [21], [22], [34]–[36]. Furthermore, hDPSC seemed to have a paracrine pro-angiogenic effect in an *in vivo* model of myocardial infarction [23]. However, the exact cellular and molecular mechanisms by which hDPSC exert their paracrine angiogenic effects, remain to be clarified. Therefore, in the present study, we identified the angiogenesis-related molecules present in both cell lysates as the secretome of hDPSC by means of a protein array and validated these results with RT-PCR and/or ELISA. As previously reported, hDPSC express high amounts of VEGF [21], [22]. This expression was not induced by culture conditions, as dental pulp tissue also showed *in situ* expression of this protein. Other pro-angiogenic factors such as IL-8 and MCP-1, which both stimulate endothelial migration [17], [37], were also detected in the cell lysates and medium. Earlier studies already mentioned the expression of both these chemotactic proteins, which were shown to be upregulated by FGF-2 [38], [39]. In this study, the pro-angiogenic factor FGF-2 was only detected in the cell lysate and not in the secretome. This was validated by means of ELISA, where FGF-2 was abundantly present in the cell lysates while the amount of FGF-2 secreted into the medium was below the detection range. In contrast, Tran-Hung *et al.* reported the presence of FGF-2 in CM of DPSC [21]. However, it should be mentioned that their CM was prepared in medium containing 10% FBS (in contrast to 0.1% FBS in our medium), while it was shown that serum increases the CM concentrations of angiogenic factors such as VEGF [40]. In line with previous reports regarding pulp stem cells from deciduous and permanent teeth, anti-angiogenic factors endostatin and IGFBP3 were also found to be expressed by hDPSC [41], [42]. In addition, uPA, TIMP-1 and PAI-1 were shown to be secreted in relatively high concentrations. As these three enzymes play a role in the stimulation or inhibition of ECM degradation, it might be useful in the future to investigate whether hDPSC can play a role in EC invasion [17], [37]. However, this could be complicated, since hDPSC express both inducers as well as inhibitors of ECM degradation. Other MSC have been shown to promote ECM breakdown and thus EC invasion. For example, in a fibrin-based *in vitro* model adipose-derived MSC and BM-MSC induced EC invasion, although both stem cell populations did this via upregulation of distinct proteases [43].

Taken together, these results show that both pro-and-anti-angiogenic factors are produced by hDPSC. Therefore, in the this study, the angiogenic capacities of the hDPSC were further investigated to assess whether the angiogenic balance of hDPSC is shifted towards blood vessel formation or towards inhibition of vascularization.

When studying the angiogenic effects of a particular stem cell population, it is not only required to identify the angiogenic molecules these cells secrete, but it is of utmost importance to investigate the effect of these stem cells on the biological behavior of EC. Since angiogenesis is a multistep process, several *in vitro* models that mimic the most important steps of angiogenesis were performed. First, the effect of the CM of hDPSC on the cell growth of two endothelial cell lines (MBEC and HMEC-1) was examined. Despite the presence of several growth factors and mitogens in the CM of hDPSC (such as VEGF), hDPSC were not able to induce cell proliferation of both cell types, while our positive control (medium with 10% FBS) significantly increased cell growth. There is a possibility that the concentrations of growth factors within the hDPSC CM is not high enough to induce EC growth. Another explanation may lie in the presence of anti-angiogenic molecules in the hDPSC CM (such as endostatin an IGFBP-3) that may interfere with and strongly inhibit the proliferative capacities of VEGF and other mitogens. Other MSC such as adipose MSC have been shown to increase EC proliferation [44]. For BM-MSC, the situation is more complicated: Gruber *et al.* reported that CM of BM-MSC had no effect on the EC proliferation [45], while others demonstrated that BM-MSC induced EC growth [40], [46]. However, the CM of the latter contained more FBS (10 and 5% respectively), while our DPSC CM has only 0.1% of FBS. As previously mentioned, FBS is able to increase the amount of angiogenic factors such as VEGF in CM [40].

Another key step in the angiogenic cascade is EC migration. In this process, EC migrate towards a gradient of angiogenic stimuli. To assess the chemotactic potential of hDPSC, an *in vitro* transwell system was used. The migration of HMEC-1 (in the upper compartment) towards hDPSC in the lower well was investigated. hDPSC significantly increased the HMEC-1 migration. This migration was inhibited by anti-VEGF antibodies, leading to the conclusion that VEGF is (at least in part) responsible for the chemotactic activities of hDPSC. However, this inhibition of HMEC-1 migration was not complete, meaning that other factors than VEGF cause the migration of HMEC-1. This factor was not MCP-1 as incubation with anti-MCP-1 alone nor a combination of anti-VEGF and anti-MCP-1 was able to fully interrupt the chemotactic activity of hDPSC. Another plausible chemotactic agent detected in the hDPSC CM is IL-8. The concentration of IL-8 in the CM is considered to be too low to cause EC migration (only 25 pg/ml) and addition of IL-8 antibodies had no effect on hDPSC-induced HMEC-1 migration (data not shown). It might be possible that VEGF acts together with other yet unidentified angiogenic molecules, as the antibody array applied in this study is only able to detect 55 different proteins, while there are far more angiogenic stimuli. In the future, it might be interesting to analyze the secretome of hDPSC with mass spectrometry to identify more angiogenic proteins, as has already been done for BM-MSC by Estrada *et al.* who discovered that the cysteine-rich protein 61 (Cyr61) was responsible for the angiogenic activities of BM-MSC [47]. Furthermore, it is also worth mentioning that not only proteins but also other molecules (such as the sugar 2′-deoxy-D-ribose) have the ability to induce EC migration [48] and that hDPSC might produce such non-protein angiogenic factors. Other MSC with chemotactic activities include Warton Jelly-derived umbilical vein MSC [49], amniotic MSC [50] and BM-MSC [45], [51], which produce high amounts of VEGF and SDF-1 [51].

In order elucidate which intracellular pathways of the HMEC-1 are activated by the hDPSC, the PI3K-inhibitor LY294002 (10 and 1 µM) and the MEK-inhibitor U0126 (10 and 1 µM) were added to the upper compartment containing the HMEC-1. Phosphatidylinositide 3-kinase (PI3K) is an intracellular signal transducer that upon activation induces phosphorylation of AKT. Phosphorylated AKT will activate gene products that promote cell survival, invasion and motility [52]. The intracellular molecule MEK is able to activate the ERK1/2 pathway which is also involved in EC survival, proliferation and migration [53]. Both LY294002 and U0126 were able to significantly reduce the hDPSC-mediated HMEC-1 migration for approximately 50% at a concentration of 10 µM, while 1 µM of these components had only a slight inhibitory effect. Addition of both LY294002 and U0126 (both 10 µM) completely abrogated HMEC-1 migration, leading to the conclusion that these inhibitors have a synergistic effect. Thus, both the PI3K/AKT and the MEK/ERK pathways are activated in the HMEC-1 by angiogenic factors secreted by the hDPSC. This is in the line with our aforementioned results where VEGF-antibodies block the chemotactic activity of hDPSC, since VEGF is able to activate both pathways by binding to its receptor [54].

In the last part of this study, we investigated whether hDPSC are able to induce blood vessel formation in a small animal model: the chicken chorioallantoic membrane (CAM) assay. The CAM is an extra-embryonic, highly vascularized tissue that serves as a transient gas exchange surface for the embryo (comparable to the lungs). The main cellular components of the CAM are endothelial cells but also mural cells such as pericytes are present. Over the past few decades, this model has been successfully used to investigate anti-angiogenic molecules for anticancer treatment. However, this assay is also suitable to assess the vascularization potential of transplanted tissues; as the early chicken embryo lacks a complete immune system mammalian xenografts can be implanted into the CAM without rejection [15]. As this model represents a rather simple and cost-effective procedure with limited ethical concerns, it has become a practical research tool to characterize tissue-engineered constructs [29]. In our study, application of only 50,000 hDPSC was enough to significantly stimulate angiogenesis in the CAM assay. The blood capillaries were found to grow radially towards the hDPSC-containing matrigel droplets. These findings suggest that the secretion of angiogenic molecules is responsible for the hDPSC-induced angiogenesis in the CAM, as the applied incubation period of 72 hours is considered to be too short to induce EC transdifferentiation of the transplanted cells [45], [55], [56]. Undifferentiated and endothelial-differentiated placental MSC also have angiogenic activities in this *in vivo*-like animal model [55]. In addition, collagen sponges soaked with concentrated CM of BM-MSC stimulated blood vessel growth in the CAM assay [45].

Taken together, although also anti-angiogenic factors such as PAI-1 and endostatin have been detected in the CM and cell lysates of hDPSC, these stem cells still have a strong pro-angiogenic potential *in vitro* and *in vivo*.

In conclusion, our study demonstrates that hDPSC are able to induce angiogenesis in a paracrine fashion. These easily accessible stem cells produce high amounts of angiogenic molecules such as VEGF and MCP-1. They also have the capacity to stimulate endothelial cell migration *in vitro* by activating the PI3K/AKT and MEK/ERK pathway of endothelial cells. Furthermore, in the *in vivo*-like CAM model hDPSC significantly induced the formation of blood vessels. These findings suggest that hDPSC may provide a suitable stem cell tool for tissue engineering and could be used as a treatment for pathologies correlated with inadequate angiogenesis such as stroke, myocardial infarction and chronic wounds [15], [20], [57], [58]. However, before any therapeutic application is possible, more elaborate research is required regarding the molecular pathways underlying the paracrine angiogenic effects of hDPSC. Furthermore, the role of hDPSC in endothelial tube formation, ECM degradation and their angiogenic behavior in a more extensive *in vivo* model remains to be elucidated.

## References

[1] Choi SH, Jung SY, Kwon SM, Baek SH (2012) Perspectives on stem cell therapy for cardiac regeneration. Advances and challenges. Circ J 76: 1307–1312. 22739079 10.1253/circj.cj-11-1479

[2] Gamie Z, Tran GT, Vyzas G, Korres N, Heliotis M, et al (2012) Stem cells combined with bone graft substitutes in skeletal tissue engineering. Expert Opin Biol Ther 12: 713–729. 22500826 10.1517/14712598.2012.679652

[3] Steinert AF, Rackwitz L, Gilbert F, Noth U, Tuan RS (2012) Concise review: the clinical application of mesenchymal stem cells for musculoskeletal regeneration: current status and perspectives. Stem Cells Transl Med 1: 237–247. 23197783 10.5966/sctm.2011-0036 PMC3659848

[4] Sasso RC, LeHuec JC, Shaffrey C (2005) Iliac crest bone graft donor site pain after anterior lumbar interbody fusion: a prospective patient satisfaction outcome assessment. J Spinal Disord Tech 18 Suppl: S77–81 10.1097/01.bsd.0000112045.36255.83 15699810

[5] Gronthos S, Mankani M, Brahim J, Robey PG, Shi S (2000) Postnatal human dental pulp stem cells (DPSCs) in vitro and in vivo. Proc Natl Acad Sci U S A 97: 13625–13630. 11087820 10.1073/pnas.240309797 PMC17626

[6] Huang GT, Yamaza T, Shea LD, Djouad F, Kuhn NZ, et al (2010) Stem/progenitor cell-mediated de novo regeneration of dental pulp with newly deposited continuous layer of dentin in an in vivo model. Tissue Eng Part A 16: 605–615. 19737072 10.1089/ten.tea.2009.0518 PMC2813150

[7] Gronthos S, Brahim J, Li W, Fisher LW, Cherman N, et al (2002) Stem cell properties of human dental pulp stem cells. J Dent Res 81: 531–535. 12147742 10.1177/154405910208100806

[8] Struys T, Moreels M, Martens W, Donders R, Wolfs E, et al (2010) Ultrastructural and Immunocytochemical Analysis of Multilineage Differentiated Human Dental Pulp- and Umbilical Cord-Derived Mesenchymal Stem Cells. Cells Tissues Organs 193: 366–378. 21124001 10.1159/000321400

[9] Arthur A, Rychkov G, Shi S, Koblar SA, Gronthos S (2008) Adult human dental pulp stem cells differentiate toward functionally active neurons under appropriate environmental cues. Stem Cells 26: 1787–1795. 18499892 10.1634/stemcells.2007-0979

[10] Kiraly M, Porcsalmy B, Pataki A, Kadar K, Jelitai M, et al (2009) Simultaneous PKC and cAMP activation induces differentiation of human dental pulp stem cells into functionally active neurons. Neurochem Int 55: 323–332. 19576521 10.1016/j.neuint.2009.03.017

[11] Ishkitiev N, Yaegaki K, Imai T, Tanaka T, Nakahara T, et al (2012) High-purity hepatic lineage differentiated from dental pulp stem cells in serum-free medium. J Endod 38: 475–480. 22414832 10.1016/j.joen.2011.12.011

[12] Papaccio G, Graziano A, d’Aquino R, Graziano MF, Pirozzi G, et al (2006) Long-term cryopreservation of dental pulp stem cells (SBP-DPSCs) and their differentiated osteoblasts: a cell source for tissue repair. J Cell Physiol 208: 319–325. 16622855 10.1002/jcp.20667

[13] Alge DL, Zhou D, Adams LL, Wyss BK, Shadday MD, et al (2010) Donor-matched comparison of dental pulp stem cells and bone marrow-derived mesenchymal stem cells in a rat model. J Tissue Eng Regen Med 4: 73–81. 19842108 10.1002/term.220 PMC2830796

[14] Karaoz E, Demircan PC, Saglam O, Aksoy A, Kaymaz F, et al (2011) Human dental pulp stem cells demonstrate better neural and epithelial stem cell properties than bone marrow-derived mesenchymal stem cells. Histochem Cell Biol 136: 455–473. 21879347 10.1007/s00418-011-0858-3

[15] Laschke MW, Harder Y, Amon M, Martin I, Farhadi J, et al (2006) Angiogenesis in tissue engineering: breathing life into constructed tissue substitutes. Tissue Eng 12: 2093–2104. 16968151 10.1089/ten.2006.12.2093

[16] Carmeliet P, Jain RK (2011) Molecular mechanisms and clinical applications of angiogenesis. Nature 473: 298–307. 21593862 10.1038/nature10144 PMC4049445

[17] Bhadada SV, Goyal BR, Patel MM (2011) Angiogenic targets for potential disorders. Fundam Clin Pharmacol 25: 29–47. 20199582 10.1111/j.1472-8206.2010.00814.x

[18] Carmeliet P, Jain RK (2011) Principles and mechanisms of vessel normalization for cancer and other angiogenic diseases. Nat Rev Drug Discov 10: 417–427. 21629292 10.1038/nrd3455

[19] Folkman J (2007) Is angiogenesis an organizing principle in biology and medicine? J Pediatr Surg 42: 1–11. 17208533 10.1016/j.jpedsurg.2006.09.048

[20] Sieveking DP, Ng MK (2009) Cell therapies for therapeutic angiogenesis: back to the bench. Vasc Med 14: 153–166. 19366823 10.1177/1358863X08098698

[21] Tran-Hung L, Laurent P, Camps J, About I (2008) Quantification of angiogenic growth factors released by human dental cells after injury. Arch Oral Biol 53: 9–13. 17764655 10.1016/j.archoralbio.2007.07.001

[22] Tran-Hung L, Mathieu S, About I (2006) Role of human pulp fibroblasts in angiogenesis. J Dent Res 85: 819–823. 16931864 10.1177/154405910608500908

[23] Gandia C, Arminan A, Garcia-Verdugo JM, Lledo E, Ruiz A, et al (2008) Human dental pulp stem cells improve left ventricular function, induce angiogenesis, and reduce infarct size in rats with acute myocardial infarction. Stem Cells 26: 638–645. 18079433 10.1634/stemcells.2007-0484

[24] Ishizaka R, Hayashi Y, Iohara K, Sugiyama M, Murakami M, et al (2013) Stimulation of angiogenesis, neurogenesis and regeneration by side population cells from dental pulp. Biomaterials 34: 1888–1897. 23245334 10.1016/j.biomaterials.2012.10.045

[25] Struys T, Ketkar-Atre A, Gervois P, Leten C, Hilkens P, et al.. (2012) Magnetic resonance imaging of human dental pulp stem cells in vitro and in vivo. Cell Transplant. 10.3727/096368912X657774 23050936

[26] Martens W, Wolfs E, Struys T, Politis C, Bronckaers A, et al (2012) Expression Pattern of Basal Markers in Human Dental Pulp Stem Cells and Tissue. Cells Tissues Organs 196: 490–500. 22739146 10.1159/000338654

[27] Bastaki M, Nelli EE, Dell’Era P, Rusnati M, Molinari-Tosatti MP, et al (1997) Basic fibroblast growth factor-induced angiogenic phenotype in mouse endothelium. A study of aortic and microvascular endothelial cell lines. Arterioscler Thromb Vasc Biol 17: 454–464. 9102163 10.1161/01.atv.17.3.454

[28] Liekens S, Bronckaers A, Belleri M, Bugatti A, Sienaert R, et al (2012) The thymidine phosphorylase inhibitor 5′-O-tritylinosine (KIN59) is an antiangiogenic multitarget fibroblast growth factor-2 antagonist. Mol Cancer Ther 11: 817–829. 22302099 10.1158/1535-7163.MCT-11-0738

[29] Baiguera S, Macchiarini P, Ribatti D (2012) Chorioallantoic membrane for in vivo investigation of tissue-engineered construct biocompatibility. J Biomed Mater Res B Appl Biomater 100: 1425–1434. 22279003 10.1002/jbm.b.32653

[30] d’Aquino R, Graziano A, Sampaolesi M, Laino G, Pirozzi G, et al (2007) Human postnatal dental pulp cells co-differentiate into osteoblasts and endotheliocytes: a pivotal synergy leading to adult bone tissue formation. Cell Death Differ 14: 1162–1171. 17347663 10.1038/sj.cdd.4402121

[31] Karbanova J, Soukup T, Suchanek J, Pytlik R, Corbeil D, et al (2011) Characterization of dental pulp stem cells from impacted third molars cultured in low serum-containing medium. Cells Tissues Organs 193: 344–365. 21071916 10.1159/000321160

[32] Marchionni C, Bonsi L, Alviano F, Lanzoni G, Di Tullio A, et al (2009) Angiogenic potential of human dental pulp stromal (stem) cells. Int J Immunopathol Pharmacol 22: 699–706. 19822086 10.1177/039463200902200315

[33] Cordeiro MM, Dong Z, Kaneko T, Zhang Z, Miyazawa M, et al (2008) Dental pulp tissue engineering with stem cells from exfoliated deciduous teeth. J Endod 34: 962–969. 18634928 10.1016/j.joen.2008.04.009

[34] Aranha AM, Zhang Z, Neiva KG, Costa CA, Hebling J, et al (2010) Hypoxia enhances the angiogenic potential of human dental pulp cells. J Endod 36: 1633–1637. 20850667 10.1016/j.joen.2010.05.013

[35] Matsushita K, Motani R, Sakuta T, Yamaguchi N, Koga T, et al (2000) The role of vascular endothelial growth factor in human dental pulp cells: induction of chemotaxis, proliferation, and differentiation and activation of the AP-1-dependent signaling pathway. J Dent Res 79: 1596–1603. 11023281 10.1177/00220345000790081201

[36] Nakashima M, Iohara K, Sugiyama M (2009) Human dental pulp stem cells with highly angiogenic and neurogenic potential for possible use in pulp regeneration. Cytokine Growth Factor Rev 20: 435–440. 19896887 10.1016/j.cytogfr.2009.10.012

[37] Distler JH, Hirth A, Kurowska-Stolarska M, Gay RE, Gay S, et al (2003) Angiogenic and angiostatic factors in the molecular control of angiogenesis. Q J Nucl Med 47: 149–161. 12897707

[38] Kim YS, Min KS, Jeong DH, Jang JH, Kim HW, et al (2010) Effects of fibroblast growth factor-2 on the expression and regulation of chemokines in human dental pulp cells. J Endod 36: 1824–1830. 20951295 10.1016/j.joen.2010.08.020

[39] Park SH, Hsiao GY, Huang GT (2004) Role of substance P and calcitonin gene-related peptide in the regulation of interleukin-8 and monocyte chemotactic protein-1 expression in human dental pulp. Int Endod J 37: 185–192. 15009408 10.1111/j.0143-2885.2004.00782.x

[40] Potapova IA, Gaudette GR, Brink PR, Robinson RB, Rosen MR, et al (2007) Mesenchymal stem cells support migration, extracellular matrix invasion, proliferation, and survival of endothelial cells in vitro. Stem Cells 25: 1761–1768. 17395769 10.1634/stemcells.2007-0022

[41] Gotz W, Heinen M, Lossdorfer S, Jager A (2006) Immunohistochemical localization of components of the insulin-like growth factor system in human permanent teeth. Arch Oral Biol 51: 387–395. 16321360 10.1016/j.archoralbio.2005.10.005

[42] Miura M, Gronthos S, Zhao M, Lu B, Fisher LW, et al (2003) SHED: stem cells from human exfoliated deciduous teeth. Proc Natl Acad Sci U S A 100: 5807–5812. 12716973 10.1073/pnas.0937635100 PMC156282

[43] Kachgal S, Putnam AJ (2011) Mesenchymal stem cells from adipose and bone marrow promote angiogenesis via distinct cytokine and protease expression mechanisms. Angiogenesis 14: 47–59. 21104120 10.1007/s10456-010-9194-9 PMC3369878

[44] Rehman J, Traktuev D, Li J, Merfeld-Clauss S, Temm-Grove CJ, et al (2004) Secretion of angiogenic and antiapoptotic factors by human adipose stromal cells. Circulation 109: 1292–1298. 14993122 10.1161/01.CIR.0000121425.42966.F1

[45] Gruber R, Kandler B, Holzmann P, Vogele-Kadletz M, Losert U, et al (2005) Bone marrow stromal cells can provide a local environment that favors migration and formation of tubular structures of endothelial cells. Tissue Eng 11: 896–903. 15998229 10.1089/ten.2005.11.896

[46] Kinnaird T, Stabile E, Burnett MS, Shou M, Lee CW, et al (2004) Local delivery of marrow-derived stromal cells augments collateral perfusion through paracrine mechanisms. Circulation 109: 1543–1549. 15023891 10.1161/01.CIR.0000124062.31102.57

[47] Estrada R, Li N, Sarojini H, An J, Lee MJ, et al (2009) Secretome from mesenchymal stem cells induces angiogenesis via Cyr61. J Cell Physiol 219: 563–571. 19170074 10.1002/jcp.21701 PMC2860629

[48] Bronckaers A, Gago F, Balzarini J, Liekens S (2009) The dual role of thymidine phosphorylase in cancer development and chemotherapy. Med Res Rev 29: 903–953. 19434693 10.1002/med.20159 PMC7168469

[49] Choi M, Lee HS, Naidansaren P, Kim HK, O E, et al (2013) Proangiogenic features of Wharton’s jelly-derived mesenchymal stromal/stem cells and their ability to form functional vessels. Int J Biochem Cell Biol 45: 560–570. 23246593 10.1016/j.biocel.2012.12.001

[50] Kim SW, Zhang HZ, Kim CE, Kim JM, Kim MH (2012) Amniotic mesenchymal stem cells with robust chemotactic properties are effective in the treatment of a myocardial infarction model. Int J Cardiol. 10.1016/j.ijcard.2012.11.003 23218573

[51] Burlacu A, Grigorescu G, Rosca AM, Preda MB, Simionescu M (2013) Factors secreted by mesenchymal stem cells and endothelial progenitor cells have complementary effects on angiogenesis in vitro. Stem Cells Dev 22: 643–653. 22947186 10.1089/scd.2012.0273 PMC3564466

[52] Osaki M, Oshimura M, Ito H (2004) PI3K-Akt pathway: its functions and alterations in human cancer. Apoptosis 9: 667–676. 15505410 10.1023/B:APPT.0000045801.15585.dd

[53] Roskoski R Jr (2012) ERK1/2 MAP kinases: structure, function, and regulation. Pharmacol Res 66: 105–143. 22569528 10.1016/j.phrs.2012.04.005

[54] Zachary I (2003) VEGF signalling: integration and multi-tasking in endothelial cell biology. Biochem Soc Trans 31: 1171–1177. 14641020 10.1042/bst0311171

[55] Lee MY, Huang JP, Chen YY, Aplin JD, Wu YH, et al (2009) Angiogenesis in differentiated placental multipotent mesenchymal stromal cells is dependent on integrin alpha5beta1. PLoS One 4: e6913. 19847290 10.1371/journal.pone.0006913 PMC2760707

[56] Talavera-Adame D, Dafoe DC, Ng TT, Wachsmann-Hogiu S, Castillo-Henkel C, et al (2009) Enhancement of embryonic stem cell differentiation promoted by avian chorioallantoic membranes. Tissue Eng Part A 15: 3193–3200. 19364272 10.1089/ten.TEA.2009.0024

[57] Chamberlain G, Fox J, Ashton B, Middleton J (2007) Concise review: mesenchymal stem cells: their phenotype, differentiation capacity, immunological features, and potential for homing. Stem Cells 25: 2739–2749. 17656645 10.1634/stemcells.2007-0197

[58] Phinney DG, Prockop DJ (2007) Concise review: mesenchymal stem/multipotent stromal cells: the state of transdifferentiation and modes of tissue repair–current views. Stem Cells 25: 2896–2902. 17901396 10.1634/stemcells.2007-0637

